# Short-term clinical efficacy and safety of unilateral biportal endoscopic transforaminal lumbar interbody fusion versus minimally invasive transforaminal lumbar interbody fusion in the treatment of lumbar degenerative diseases: a systematic review and meta-analysis

**DOI:** 10.1186/s13018-023-04138-0

**Published:** 2023-09-04

**Authors:** Hao Han, Yifan Song, Yiming Li, Hengcai Zhou, Yufei Fu, Jie Li

**Affiliations:** 1 Graduate School of Xuzhou Medical University, Xuzhou, Jiangsu China; 2 Graduate School of Bengbu Medical College, Bengbu, Anhui China; 3 Present Address: Department of Orthopaedics, Xuzhou Central Hospital, Affiliation Xuzhou Clinical College of Xuzhou Medical University, Jiefang South Road No. 199, Xuzhou, 221009 Jiangsu China; 4 Department of Medical Imaging, Xuzhou Central Hospital, Xuzhou Clinical College of Xuzhou Medical University, Jiefang South Road No. 199, Xuzhou, 221009 Jiangsu China

**Keywords:** Unilateral biportal endoscopic, Minimally invasive spine surgery, Lumbar interbody fusion, Meta-analysis

## Abstract

**Background:**

The aim of this study was to comprehensively evaluate the short-term clinical efficacy and safety of unilateral biportal endoscopic transforaminal lumbar interbody fusion (UBE-TLIF) versus minimally invasive transforaminal lumbar interbody fusion (MIS-TLIF) for the treatment of lumbar degenerative diseases by meta-analysis.

**Methods:**

A computer-based search of PubMed, Embase, Web of Science, Cochrane Database, China National Knowledge Infrastructure (CNKI), Wanfang Database, and Chinese Science and Technology Journal Database (VIP) was conducted from the inception of the each database to April 2023. The searched literature was then screened according to strict inclusion and exclusion criteria. The critical data were extracted and analyzed using Review Manager software5.4.1. Pooled effects were calculated on the basis of data attributes by mean difference (MD) or odds ratio (OR) with 95% confidence interval (CI). The Newcastle–Ottawa Scale was used to assess the quality of the studies.

**Results:**

A total of 13 studies and 949 patients met the inclusion criteria for this meta-analysis, 445 in the UBE-LIF group and 504 in the MIS-TLIF group. UBE-TLIF was superior to MIS-TLIF in terms of intraoperative blood flow, postoperative drainage flow, duration of hospital stay, VAS score for low back pain and ODI score, but the operative time was longer than MIS-TLIF group. There were no significant differences between the two groups in terms of total complication rate, modified Macnab grading criteria, fusion rate, VAS score of leg pain, lumbar lordosis, intervertebral disk height.

**Conclusion:**

Both UBE-TLIF and MIS-TLIF are effective surgical modalities for the treatment of degenerative lumbar spine diseases. They have similar treatment outcomes, but UBE-TLIF has the advantages of less intraoperative blood loss, shorter postoperative hospital stay, and faster recovery.

*Trial registration*: This study has been registered at INPLASY.COM (No. INPLASY202320087).

**Supplementary Information:**

The online version contains supplementary material available at 10.1186/s13018-023-04138-0.

## Introduction

Lumbar degenerative disease is a common disease occurring in the elderly population, which mainly includes lumbar disk herniation, lumbar spinal stenosis, lumbar degenerative spondylolisthesis, degenerative scoliosis, etc. It results in structural instability, sciatica, radiating discomfort to the lower limbs, and low back pain [1, 2]. Lumbar interbody fusion is an effective treatment option for lumbar degenerative disease, which decompresses the spinal canal and restores structural stability to the spine [3]. Transforaminal lumbar interbody fusion (TLIF) is a procedure used to treat conditions of the lumbar spine because it reduces the strain on the dural capsule and nerve roots, reducing the incidence of associated complications and is therefore used in the treatment of lumbar spine disorders [4, 5]. Foley et al. [6] have reported a minimally invasive TLIF (MIS-TLIF) technique using a tubular retractor and microscope, which reduces iatrogenic induced injuries to soft tissues such as paravertebral muscles, with less trauma, less blood loss, and faster postoperative recovery [7, 8]. However, MIS-TLIF also has its disadvantages, especially for deeper surgical areas, the surgical field of view of MIS-TLIF is not sufficiently clear, which brings some challenges to the surgical operation, and the tubular retractor used to pull the paravertebral muscles leading to local soft tissue ischemia, which affects the postoperative recovery. The unilateral biportal endoscopic (UBE) approach has been initially described by De Antoni et al. [9]. The application of UBE to lumbar spine fusion has been firstly reported by Heo et al. in 2017 and excellent clinical results [10]. More and more surgeons are coming to recognize UBE as a safe, efficient, and different minimally invasive procedure. It uses two mutually independent channels for visualization and operation, combining the benefits of wide visualization and flexible operation in conventional open surgery and minimal tissue damage and quick postoperative recovery in minimally invasive surgery [11–13]. The present study has conducted to systematically evaluate the efficacy and safety of UBE-TLIF and MIS-TLIF in the treatment of degenerative lumbar spine diseases and to provide reference values for clinical application.

## Materials and methods

### Search strategy

A comprehensive search of PubMed, Embase, Web of Science, Cochrane Database, China National Knowledge Infrastructure (CNKI), Wanfang Database, and Chinese Science and Technology Journal Database (VIP) was conducted from the inception of the each database to April 2023. The search keywords were “unilateral biportal endoscopic lumbar interbody fusion,” “biportal endoscopic lumbar interbody fusion,” “BE-LIF,” “UBE-LIF,” “ULIF” “minimally invasive transforaminal lumbar interbody fusion,” “MIS-TLIF.” In order to search the relevant literature as comprehensively as possible, two researchers independently screened the literature and extracted data based on the search criteria and content. When there was disagreement, the disagreement is resolved through discussion or consultation with a third researcher. The search language was limited to Chinese or English. This study has been registered at INPLASY.COM (No. INPLASY202320087).

### Inclusion and exclusion criteria for the study

The inclusion criteria were as follows: (1) contrastive study that compared UBE-TLIF with MIS-TLIF for the treatment of LDD. (2) Study designs include prospective cohort studies, retrospective studies and randomized controlled trials. (3) The search language was limited to Chinese or English. (4) Postoperative follow-up included at least two of the following reference indicators: operation time, intraoperative blood loss, postoperative drainage flow postoperative hospital stay, complication rate, modified Macnab grading criteria, fusion rate, visual analog scale (VAS) back or leg score, Oswestry Disability Index (ODI).

The exclusion criteria were as follows: (1) Non-clinical comparison studies. (2) Patients with a history of spine surgery spinal infections, tumors, rheumatic immune diseases. (3) Duplicated studies. (4) Meta-analysis, literature review, case-report, conference presentation, degree dissertation, etc. (5) Studies where data could not be extracted (Table 1).

**Table 1:** Summary of included study characteristics and outcomes

Studies (author and year)	Design	Country	No. of patients		Gender (male/female)		Age (years)		Follow-up (months)		Outcomes	NOS
			UBE-TLIF	MIS-TLIF	UBE-TLIF	MIS-TLIF	UBE-TLIF	MIS-TLIF	UBE-TLIF	MIS-TLIF		
Heo et al. 2019	Retrospective	Korea	23	46	7/16	19/27	61.4 ± 9.4	63.5 ± 10.5	13.4 ± 2.5		①②⑤⑦⑧⑨	6
Kang et al. 2021	Retrospective	Korea	47	32	17/30	17/15	66.87 ± 10.41	66.38 ± 9.45	14.5 ± 2.3	15.78 ± 3.16	①②③④⑤⑦	7
Kim et al. 2021	Retrospective	Korea	32	55	17/15	25/30	70.5 ± 8.26	67.3 ± 10.7	27.2 ± 5.4	21.5 ± 7.3	①④⑤⑥⑦⑧⑨	8
Zhu et al. 2021	Retrospective	China	35	41	16/19	19/22	50.94 ± 12.12	53.44 ± 14.37	15.29 ± 1.98	16.12 ± 2.59	①②④⑤⑥⑦⑧⑨	8
Kong et al. 2022	Retrospective	China	35	40	13/22	18/22	55.1	56.0	14.7 ± 2.5	15.0 ± 3.4	①②④⑤⑧⑨⑩⑪	7
Jiang et al. 2022	Retrospective	China	25	25	9/16	8/17	63.28 ± 8.51	59.68 ± 10.38	NA		①②③④⑫	6
Huang et al. 2022	Retrospective	China	38	44	22/16	26/18	60.16 ± 7.36	59.68 ± 6.94	12		①②③④⑤⑥⑦⑧⑨⑫	8
Gatam et al.2021	Retrospective	Indonesia	72	73	26/46	28/45	55.1 ± 5.12	52.3 ± 6.13	12		⑤⑦	6
Song et al. 2023	Retrospective	China	25	24	9/16	8/16	52.36 ± 10.69	56.38 ± 10.53	14.04 ± 1.51	14.79 ± 1.59	①②③⑤⑥⑧⑨	8
Yu et al. 2023	Retrospective	China	23	18	11/12	8/10	60.80	60.70	38.96 ± 3.17	39.5 ± 3.35	①②④⑤⑦⑨⑩⑪	8
Yang et al. 2023	Retrospective	China	30	35	12/18	20/15	49.3 ± 3.5	59.3 ± 3.6	6		①③④⑤⑥⑦	7
Ma et al.2022	Retrospective	China	32	43	19/13	26/17	58.81 ± 12.49	57.42 ± 9.67	8.2		①②③④⑤⑥⑦⑨	8
Song et al. 2022	Retrospective	China	28	28	18/38		55.5 ± 10.7		14.1 ± 1.5	14.3 ± 1.4	①②⑤⑥⑦⑧⑨⑩⑫	8

Merger of narrative: ① operative time; ② intraoperative blood loss; ③ postoperative drainage flow; ④ duration of hospital stay; ⑤ total complication rate; ⑥ modified Macnab grading criteria; ⑦ fusion rate; ⑧ VAS; ⑨ ODI; ⑩ lumbar lordosis; ⑪ intervertebral disk height; ⑫ postoperative CRP level; *NOS* Newcastle–Ottawa Scale, *NA* not available

### Data extraction

Two researchers independently extracted data from the literature. The following parameters were collected using standardized forms: (1) basic characteristics of the included study and population: author, publication year, study design, country of origin, number of patients, gender, age, follow-up time, and main outcome indicators. (2) Perioperative outcomes: including operation time, intraoperative blood loss, postoperative drainage flow, postoperative hospital stay. (3) Functional outcomes at preoperative and at last follow-up, including Oswestry Disability Index (ODI), Visual Analog Scale (VAS)-back pain, VAS-leg pain, modified Macnab grading criteria, fusion rate, lumbar lordosis (LL), Intervertebral disk height(IDH). (4) Surgical complications and outcomes at the last follow-up: total complications rate, transient palsy rate, postoperative epidural hematoma rate, dural tear rate, infection rate, incomplete decompression rate, Deep Vein Thrombosis (DVT) rate.

### Quality assessment of included studies

Two researchers independently reviewed the titles, abstracts, and full texts of the included studies (if necessary) and used the Newcastle–Ottawa Scale (NOS) to assess the quality of the included non-randomized studies. Each study was assessed by the three respects: selection of the study population, comparability of groups, and measurement of exposure factors in Table 2. Studies with scores above 5 were included in the analysis. All divergences are resolved through negotiation.

**Table 2:** Quality assessment of included retrospective studies based on the Newcastle–Ottawa Scale

Studies	Selection of the study population	Comparability of groups	Measurement of exposure factors	Quality assessment
Heo et al. 2019	2	2	2	6
Kang et al. 2021	2	3	2	7
Kim et al. 2021	3	2	3	8
Zhu et al. 2021	3	2	3	8
Kong et al. 2022	2	3	3	7
Jiang et al. 2022	2	2	2	6
Huang et al. 2022	3	2	3	8
Gatam et al.2021	2	3	1	6
Song et al. 2023	3	2	3	8
Yu et al. 2023	3	3	2	8
Yang et al. 2023	2	3	2	7
Ma et al.2022	2	2	3	8
Song et al. 2022	3	3	3	8

### Statistical analysis

The program Review Manager 5.4.1 (The Nordic Cochrane Centre, The Cochrane Collaboration) was used for statistical analysis of the pooled data. For continuous variables were analyzed using mean difference (MD) and 95% confidence interval (CI) as effect sizes. For dichotomous variables were analyzed using mean difference (MD) and 95% confidence interval (CI) as effect sizes. For dichotomous variables were analyzed using the odds ratio (OR) index and 95% confidence interval (CI) as effect sizes. *P* < 0.05 indicated that the difference was statistically significant. The heterogeneity in the results between the studies was analyzed using *Q*-test and *I*^2^ test. If *I*^2^ ≥ 50% and *P* < 0.1 indicate significant heterogeneity, a random-effects model was used for Meta-analysis; if *I*^2^ ≤ 50% and *P* > 0.1 indicate insignificant heterogeneity, a fixed-effects model was used for meta-analysis.

## Results

### Study search and selection results

**Fig. 1:**
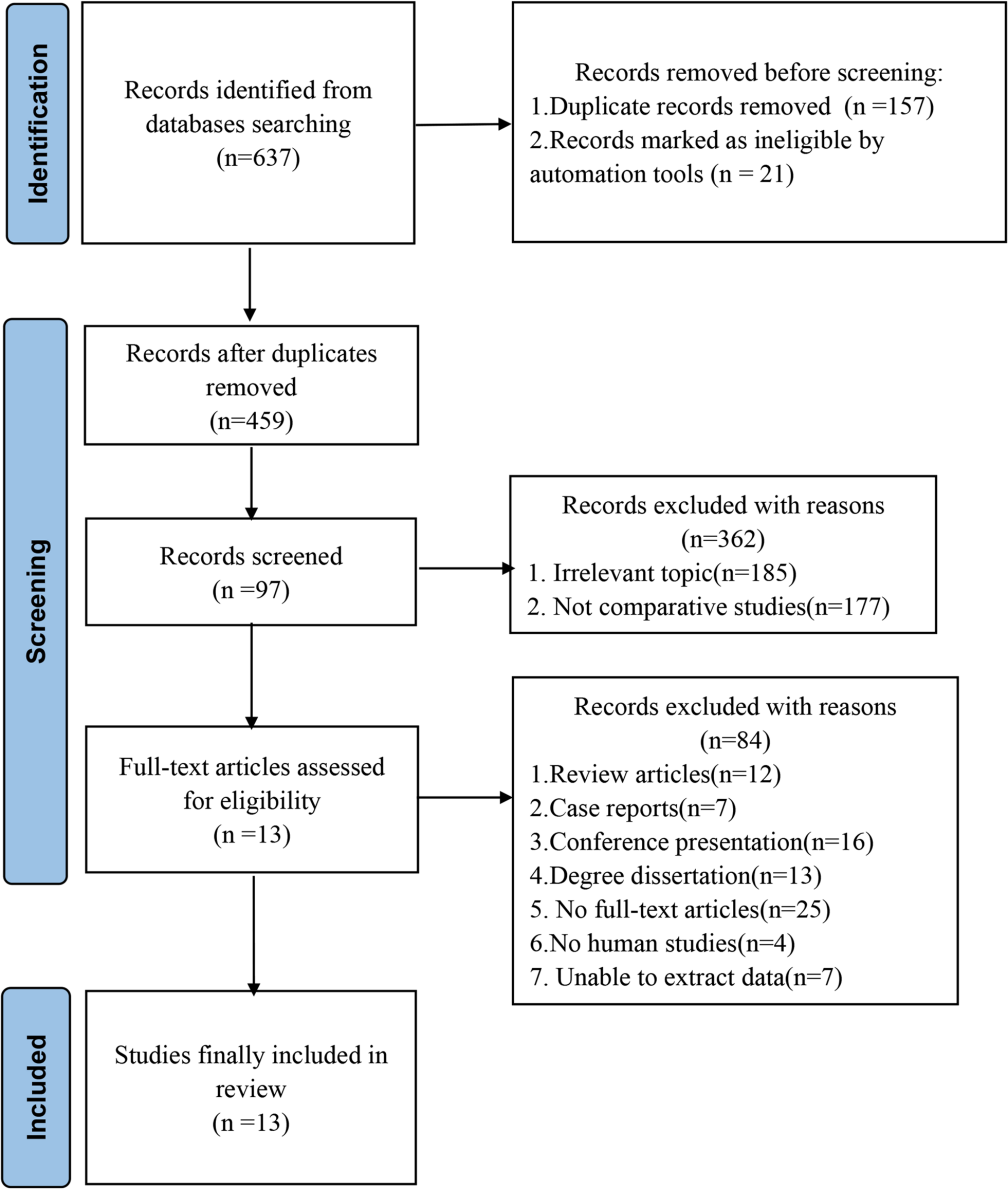
Meta-analysis flow diagram

The diagram of the literature selection process is summarized in Fig. 1. A total of 637 [14–26] studies were searched and screened through 7 databases, and 13 studies (6 literatures in English and 7 literatures in Chinese) were finally included according to the inclusion and exclusion criteria, and all included studies were retrospective case–control studies. The basic characteristics and NOS scores of the included studies are indicated in Table 1. A total of 949 patients were included, including 445 patients in the UBE-LIF group and 504 patients in the MIS-TLIF group. The year of publication of the literature was focused on 2019 to 2023. Specific complications are summarized in Table 3. This study followed the PRISMA 2020 checklist as provided in Additional file 1.

**Table 3:** Complications reported in the studies included in the Meta-analysis

Complications	UBE-TLIF (*n* = 420)	MIS-TLIF (*n* = 479)
Transient palsy	3 (0.71%)	10 (2.09%)
Postoperative epidural haematoma	5 (1.19%)	6 (1.25%)
Dural tear	14 (3.33%)	7 (1.46%)
Infection	0	7 (1.46%)
Incomplete decompression	1 (0.24%)	2 (0.42%)
DVT	0	1 (0.21%)
Cage displacement	2 (0.48%)	4 (0.84%)
Transient muscletransient muscle strength decline	1 (0.24%)	1 (0.21%)
Nerve strain injury	1 (0.24%)	1 (0.21%)
Total	27 (6.43%)	39 (8.14%)

*DVT* deep vein thrombosis

### Clinical results

#### Operative time

**Fig. 2:**
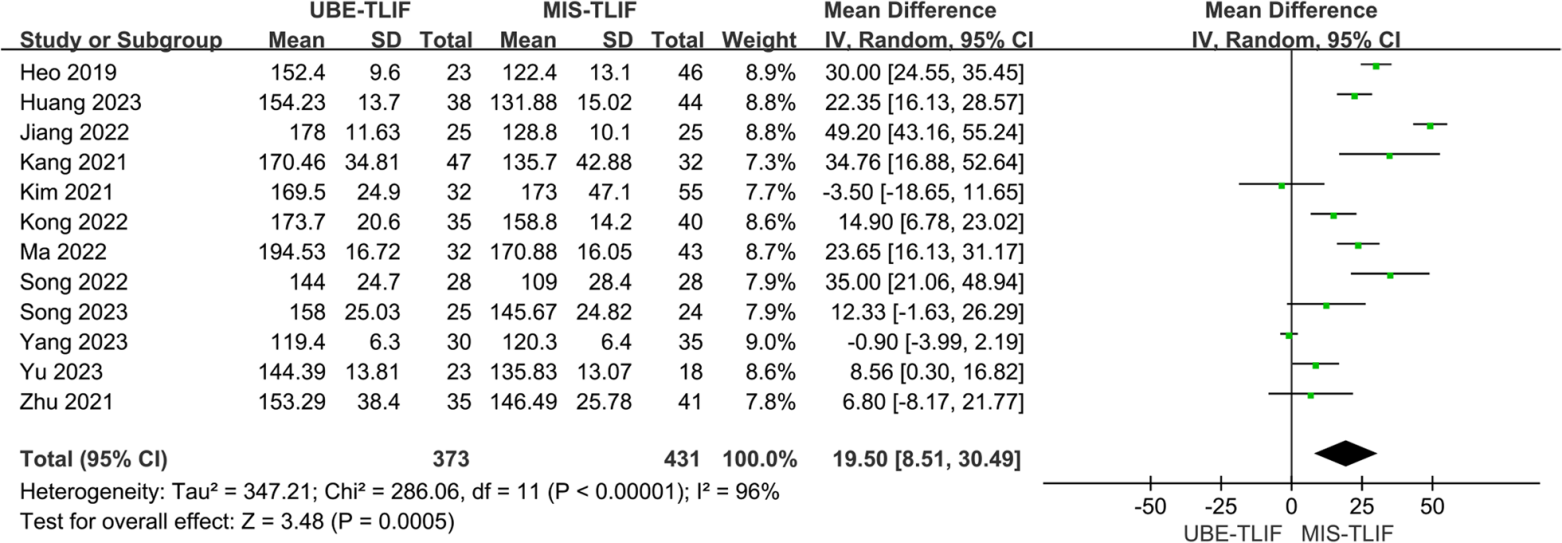
Forest plot for operative time

The operative time was reported in all 12 included studies [14, 16–26]. A total of 814 patients were included, including 373 patients in the UBE-TLIF group and 431 patients in the MIS-TLIF group. Heterogeneity between the two groups was high. (*P* < 0.00001, *I*^2^ = 96%) and a random-effect model was used for meta-analysis. The analysis result showed a statistically significant difference in operative time between the two groups, with the UBE-TLIF group having a significantly shorter operative time compared to the MIS-TLIF group. (MD = 19.5; 95% CI (8.51, 30.49); *P* = 0.0005, Fig. 2).

#### Intraoperative blood loss

**Fig. 3:**
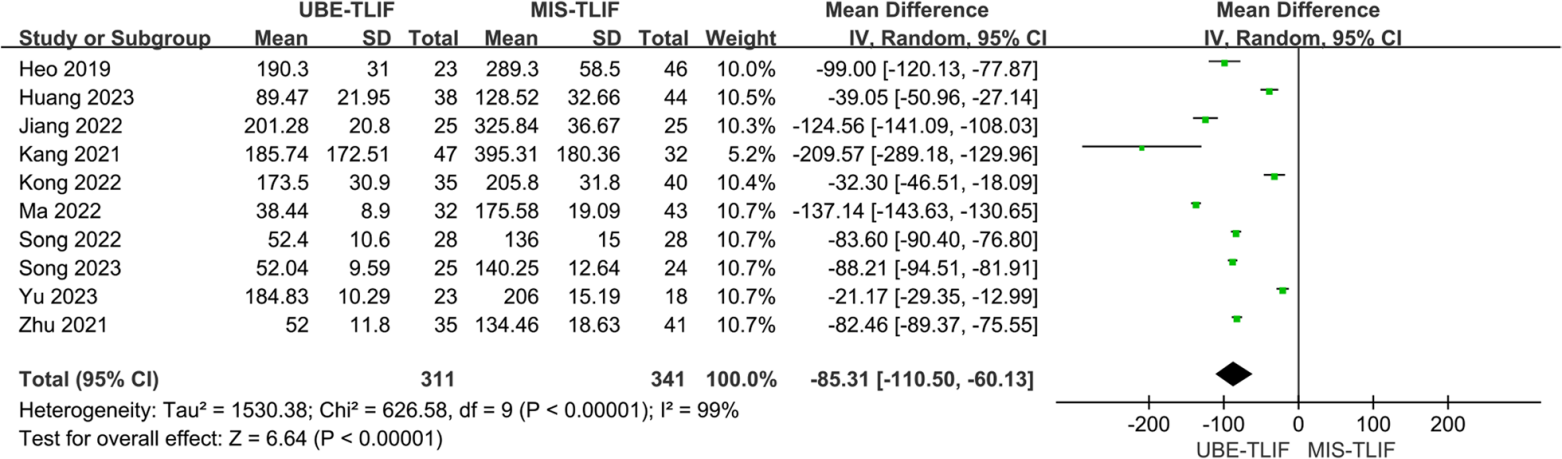
Forest plot for intraoperative blood loss

Intraoperative blood loss was reported in 10 studies [14, 16, 18–25], which included 652 patients, including 311 patients in the UBE-TLIF group and 341 patients in the MIS-TLIF group. Heterogeneity between the two groups was high. (*P* < 0.00001, *I*^2^ = 99%) and a random-effect model was used for Meta-analysis. The analysis result showed a statistically significant difference in intraoperative blood loss between the two groups, with a significantly less intraoperative blood loss in the UBE-TLIF group compared to the MIS-TLIF group (MD = − 85.31; 95% CI (− 110.50, − 60.13); *P* < 0.00001, Fig. 3).

#### Postoperative drainage flow

**Fig. 4:**
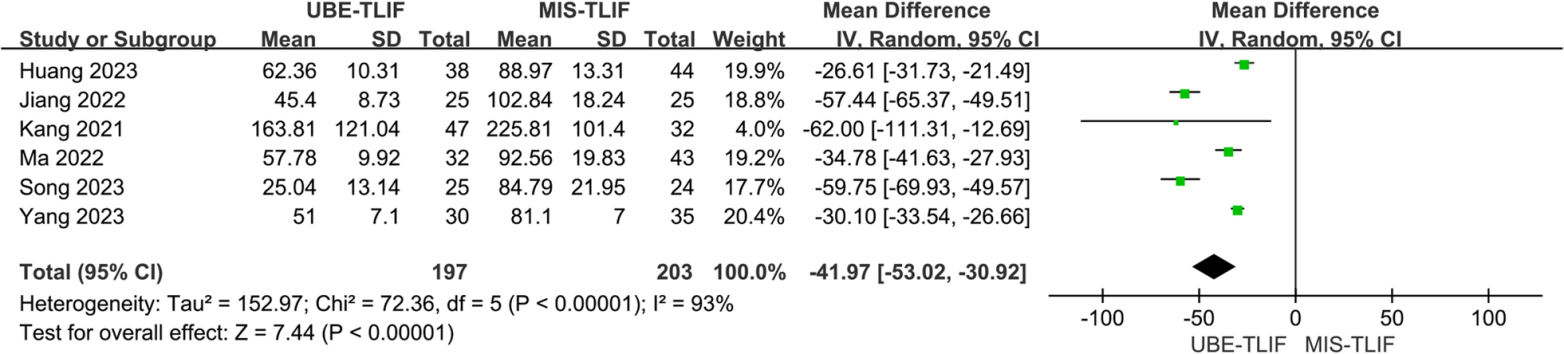
Forest plot for postoperative drainage flow

A total of 6 studies both reported postoperative drainage [16, 19, 21, 23, 24, 26]. A total of 400 patients were included, including 197 patients in the UBE-TLIF group and 203 patients in the MIS-TLIF group. Heterogeneity between the two groups was low (*P* < 0.00001, *I*^2^ = 93%), and Meta-analysis was performed using a fixed-effect model. The analysis showed no statistically significant difference in postoperative drainage between the two groups. (MD = − 41.97; 95% CI (− 53.02, − 30.92); *P* < 0.00001, Fig. 4).

### Duration of hospital stay

**Fig. 5:**
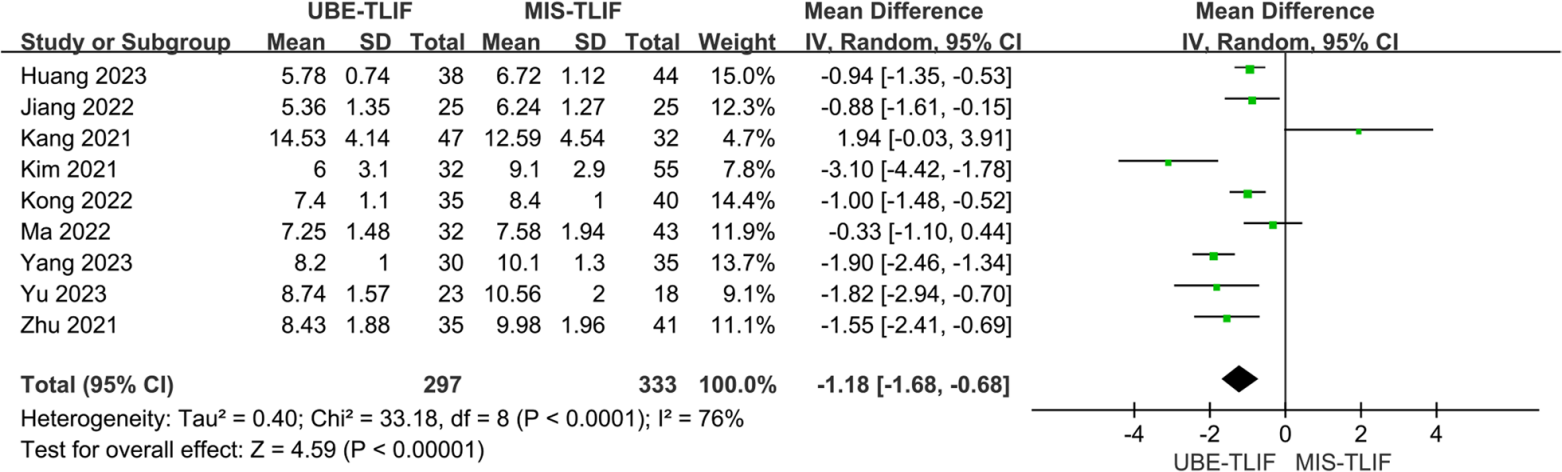
Forest plot for duration of hospital stay

A total of 9 studies reported the duration of hospital stay [16–21, 23, 25, 26]. Six hundred and thirty patients were included, including 297 patients in the UBE-TLIF group and 333 patients in the MIS-TLIF group. Heterogeneity between the two groups was high. (*P* < 0.0001, *I*^2^ = 76%) and a random-effect model was used for Meta-analysis. The analysis result showed a statistically significant difference in duration of hospital stay between the two groups, with the UBE-TLIF group having a significantly shorter postoperative hospital stay compared to the MIS-TLIF group. (MD = − 1.18; 95% CI (− 1.68; − 0.68); *P* < 0.00001, Fig. 5).

### Total complication rate

**Fig. 6:**
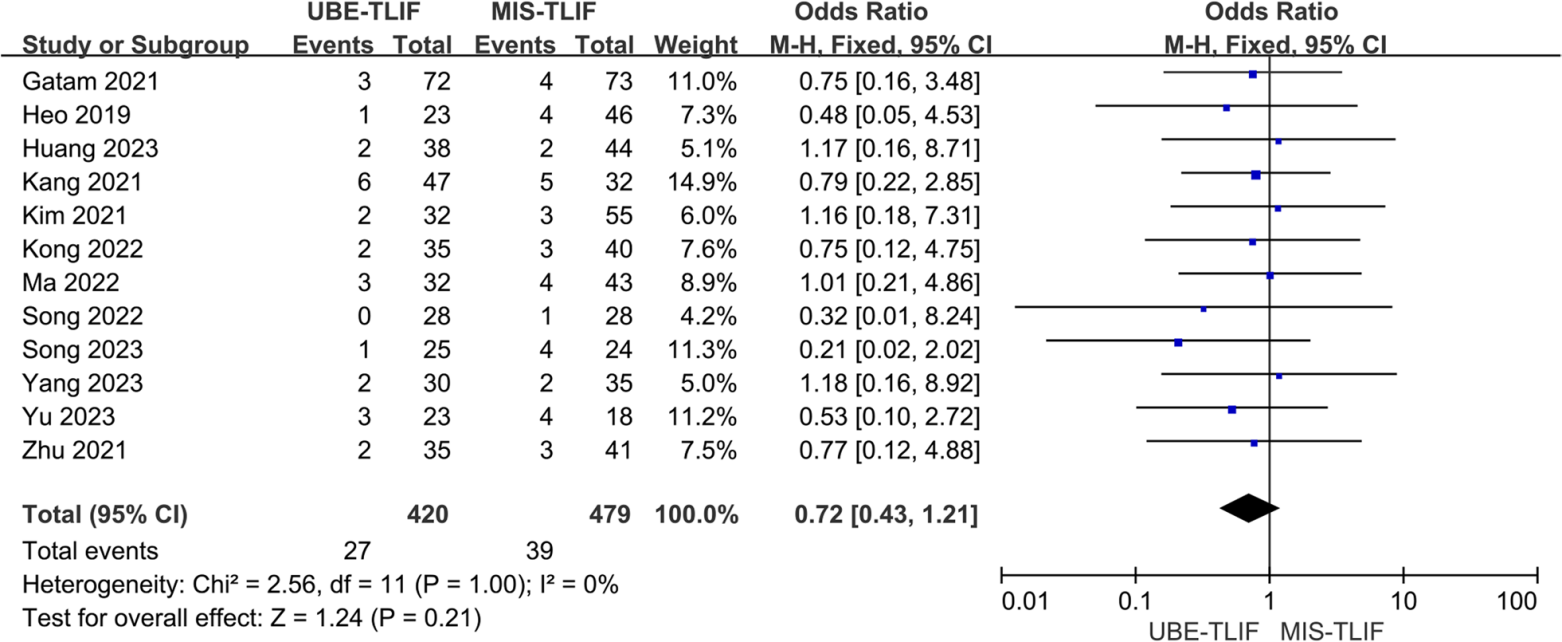
Forest plot for total complication rate

Complications were reported in a total of 12 studies [14–18, 20–26]. A total of 899 patients were included, including 420 patients in the UBE-TLIF group and 479 patients in the MIS-TLIF group. There was low heterogeneity between the two groups. (*P* = 1.00, *I*^2^ = 0%), and Meta-analysis was performed using a fixed-effect model. The analysis result showed that there was no statistically significant difference in complication rate between the two groups. (MD = 0.72; 95% CI (0.43, 1.21); *P* = 0.21, Fig. 6).

### Modified Macnab grading criteria

**Fig. 7:**
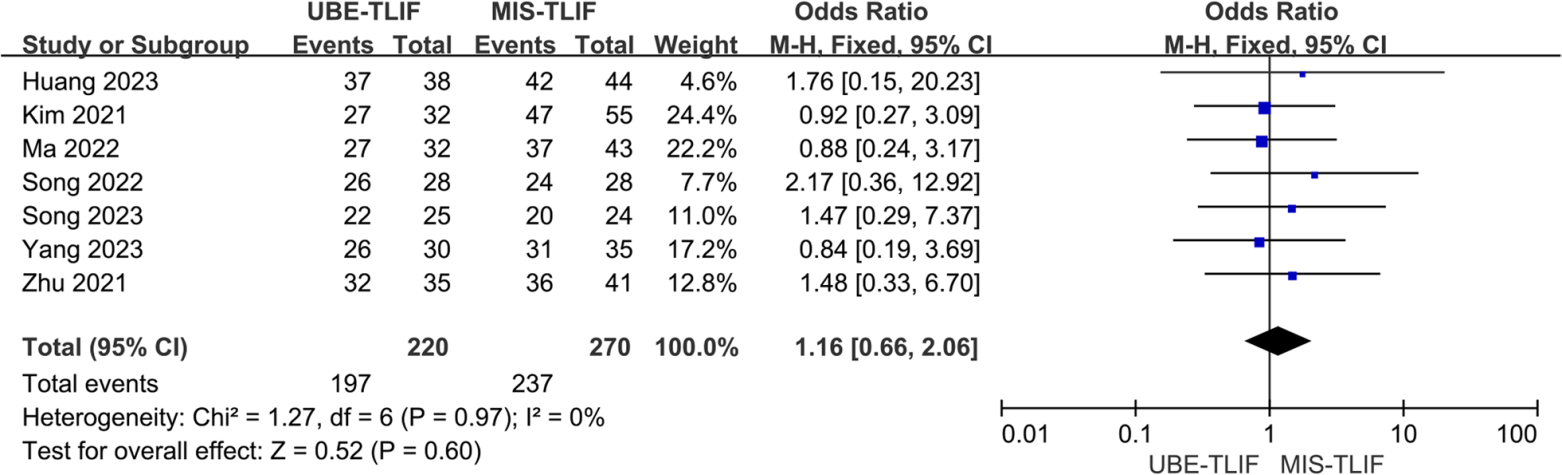
Forest plot for modified Macnab grading criteria

A total of 7 studies reported the modified Macnab grading criteria [17, 18, 21–24, 26]. A total of 490 patients were included, including 220 patients in the UBE-TLIF group and 270 patients in the MIS-TLIF group. There was low heterogeneity between the two groups (*P* = 0.97, *I*^2^ = 0%) and a fixed-effect model was used for Meta-analysis. The result of the analysis showed that there was no statistically significant difference in the Modified Macnab grading criteria between the two groups. (MD = 1.16; 95% CI (0.66, 2.06); *P* = 0.60, Fig. 7).

### Fusion rate

**Fig. 8:**
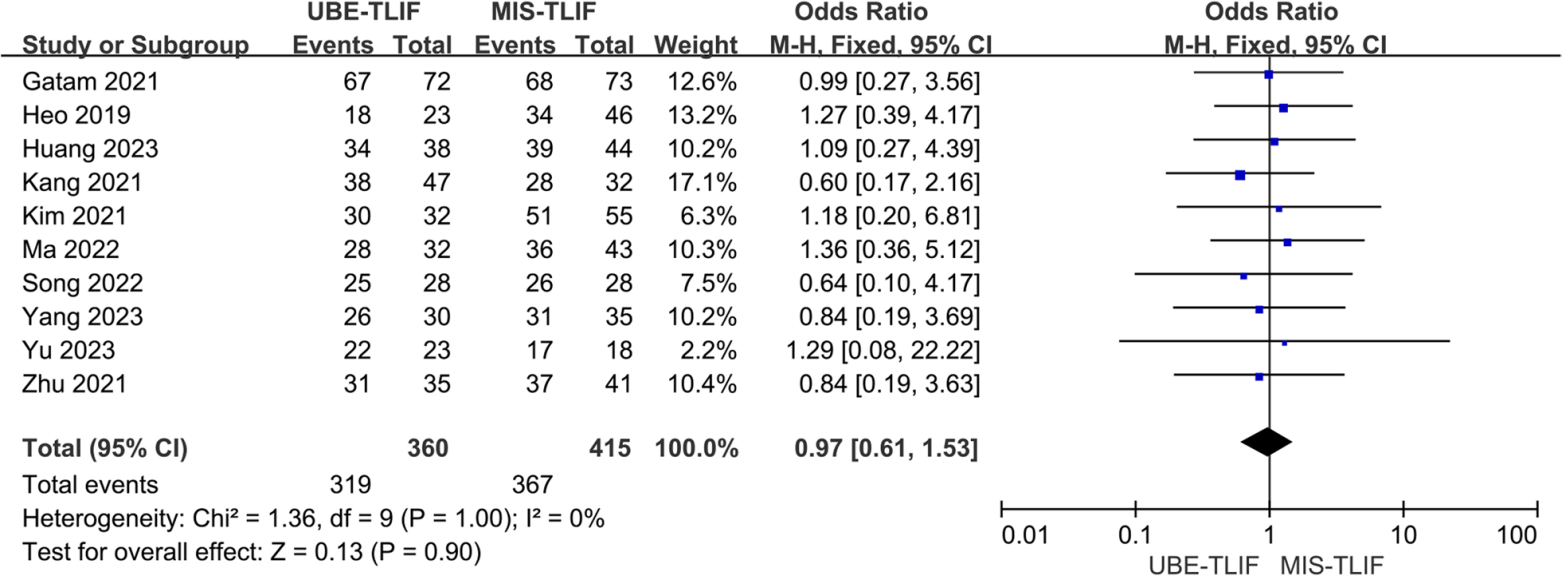
Forest plot for fusion rate

Fusion rate was reported in all 10 studies [14–18, 21–23, 25, 26]. A total of 775 patients were included, including 360 patients in the UBE-TLIF group and 415 patients in the MIS-TLIF group. Heterogeneity between the two groups was low (*P* = 1.00, *I*^2^ = 0%), and Meta-analysis was performed using a fixed-effect model. The analysis result showed no statistically significant difference in the fusion rate between the two groups. (MD = 0.97; 95% CI (0.61, 1.53); *P* = 0.90, Fig. 8).

### Postoperative low back pain VAS score (with 1 mth)

**Fig. 9:**
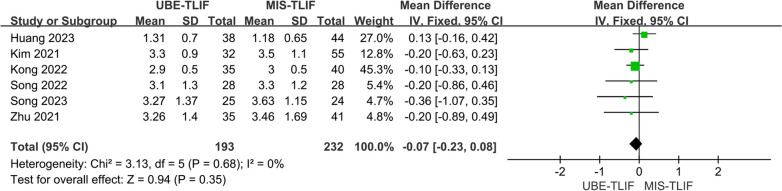
Forest plot for early postoperative low back pain VAS score

A total of 6 studies reported early postoperative low back pain VSA score [17, 18, 20, 22–24]. A total of 425 patients were included, including 193 patients in the UBE-TLIF group and 232 patients in the MIS-TLIF group. Heterogeneity between the two groups was extremely low. (*P* < 0.00001, *I*^2^ = 91%), and meta-analysis was performed using a random-effect model. The analysis result showed no statistically significant difference in VAS of low back pain at last follow-up between the two groups. (MD = − 0.96; 95% CI (− 1.57, − 0.35); *P* = 0.002, Fig. 9).

### VAS score of low back pain with 12mths

**Fig. 10:**
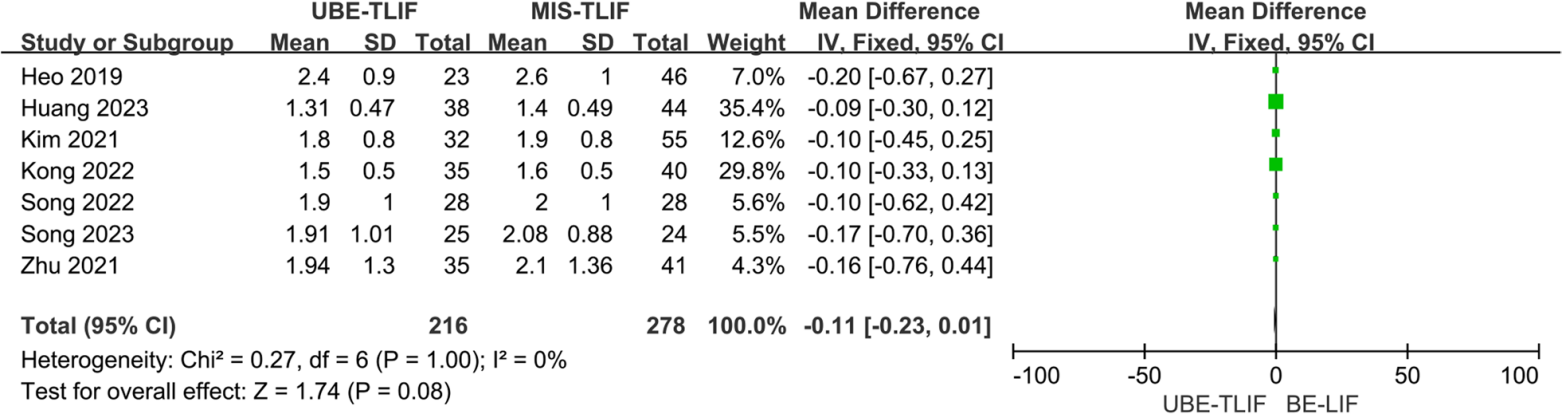
Forest plot for VAS score of low back pain at the last follow-up

A total of 7 studies reported VAS for low back pain at the last follow-up (> 12 mths) [14, 17, 18, 20, 22–24]. A total of 494 patients were included, including 216 patients in the UBE-TLIF group and 278 patients in the MIS-TLIF group. Heterogeneity between the two groups was extremely low. (*P* = 1.00, *I*^2^ = 0%), and meta-analysis was performed using a fixed-effect model. The analysis result showed no statistically significant difference in VAS of low back pain at last follow-up between the two groups. (MD = − 0.11; 95% CI (− 0.23, 0.01); *P* = 0.08, Fig. 10).

### Postoperative leg pain VSA score with 1mth

**Fig. 11:**
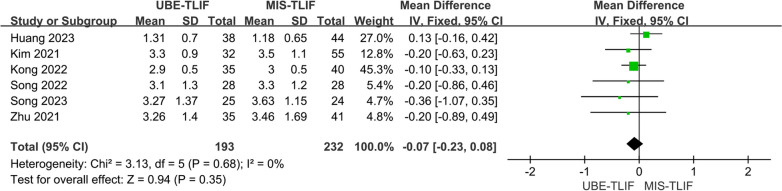
Forest plot for early postoperative leg pain VAS score. lumbar lordosis

A total of 6 studies reported early postoperative leg pain VSA score [17, 18, 20, 22–24]. A total of 425 patients were included, including 193 patients in the UBE-TLIF group and 232 patients in the MIS-TLIF group. Heterogeneity between the two groups was extremely low. (*P* = 0.68, *I*^2^ = 0%), and Meta-analysis was performed using a fixed-effect model. The analysis result showed no statistically significant difference in VAS of low back pain at last follow-up between the two groups. (MD = − 0.07; 95% CI (− 0.23, 0.08); *P* = 0.35, Fig. 11).

### VAS score of leg pain with 12 mths

**Fig. 12:**
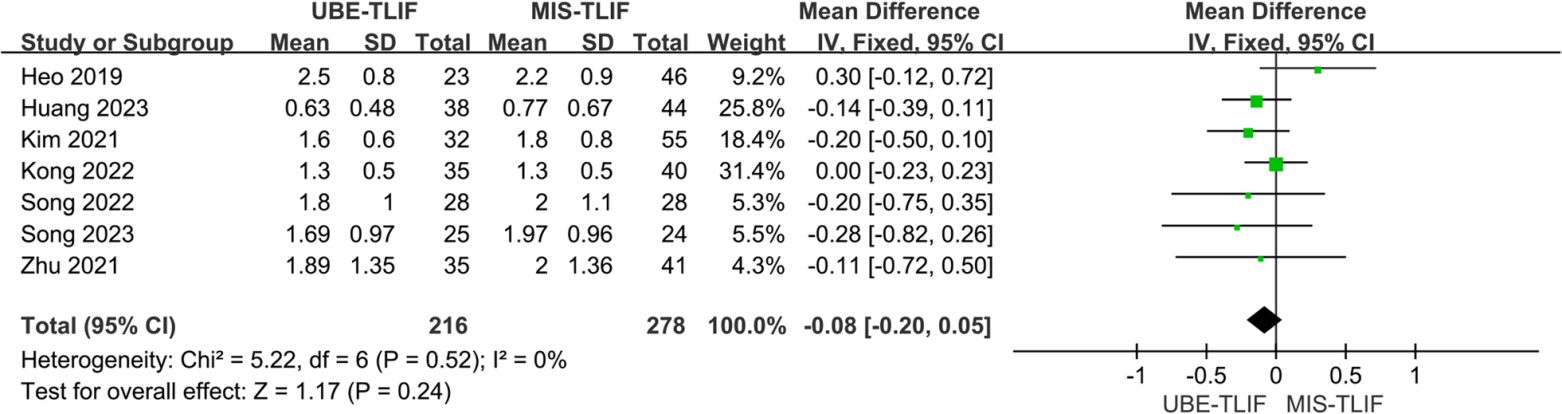
Forest plot for VAS score of leg pain at the last follow-up

A total of 7 studies reported VAS for leg pain at the last follow-up [14, 17, 18, 20, 22–24]. A total of 494 patients were included, including 216 patients in the UBE-TLIF group and 278 patients in the MIS-TLIF group. Heterogeneity between the two groups was low. (*P* = 0.52, *I*^2^ = 0%), and Meta-analysis was performed using a fixed-effect model. The analysis showed no statistically significant difference in VAS of leg pain at the last follow-up between the two groups. (MD = − 0.08; 95% CI (− 0.20, 0.05); *P* = 0.24, Fig. 12).

### ODI score with 1 mth

**Fig. 13:**
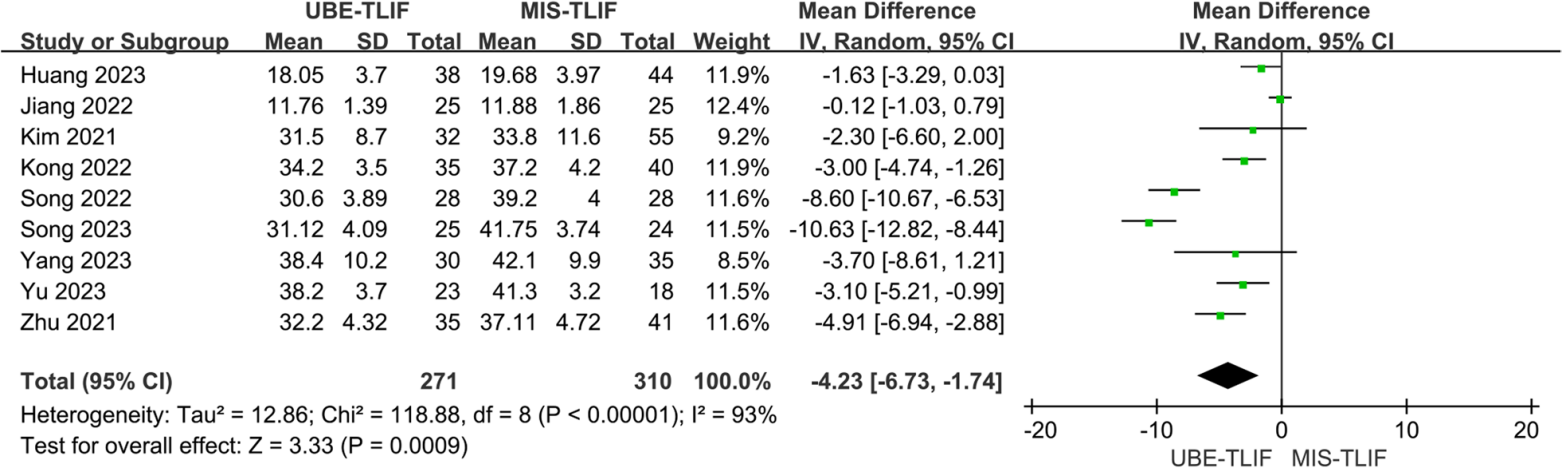
Forest plot for early ODI score

A total of 9 studies reported early ODI score [17–20, 22–26]. A total of 581 patients were included, including 271 patients in the UBE-TLIF group and 310 patients in the MIS-TLIF group. Heterogeneity between the two groups was low. (*P* < 0.00001, *I*^2^ = 93%), and Meta-analysis was performed using a fixed-effect model. The analysis result showed a statistically significant difference at the last follow-up ODI between the two groups (MD = − 4.23; 95% CI (− 6.73, − 1.74); *P* = 0.0009, Fig. 13). The ODI was significantly lower in the UBE-LIF group compared to the MIS-TLIF group.

### ODI score with 12 mths

**Fig. 14:**
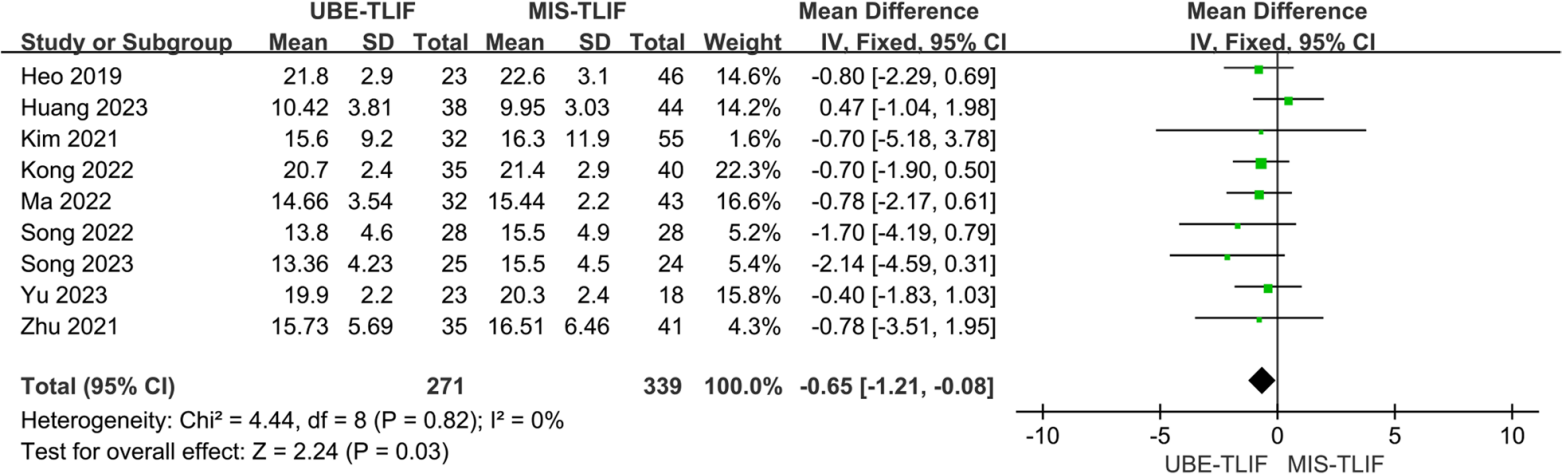
Forest plot for ODI score at the last follow-up

A total of 9 studies reported ODI score at the last follow-up [14, 17, 18, 20–25]. A total of 610 patients were included, including 271 patients in the UBE-TLIF group and 339 patients in the MIS-TLIF group. Heterogeneity between the two groups was low. (*P* = 0.82, *I*^2^ = 0%), and meta-analysis was performed using a fixed-effect model. The analysis result showed a statistically significant difference at the last follow-up ODI between the two groups (MD = − 0.65; 95% CI (− 1.21, − 0.08); *P* = 0.03, Fig. 14). The ODI was significantly lower in the UBE-TLIF group compared to the MIS-TLIF group.

### Postoperative lumbar lordosis

**Fig. 15:**
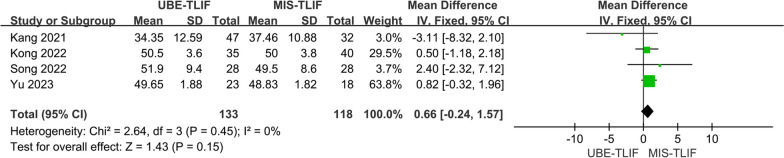
Forest plot for postoperative lumbar lordosis

A total of 4 studies both reported the postoperative lumbar lordosis (LL) [20, 22, 25]. A total of 251 patients were included, including 133 patients in the UBE-TLIF group and 118 patients in the MIS-TLIF group. Heterogeneity between the two groups was low (*P* = 0.45, *I*^2^ = 0%), and meta-analysis was performed using a fixed-effect model. The results of the analysis showed no statistically significant difference in the height of lumbar lordosis (LL) at the last follow-up between the two groups. (MD = 0.66; 95% CI (− 0.44, 1.57); *P* = 0.15, Fig. 15).

### Postoperative intervertebral disk height

**Fig. 16:**
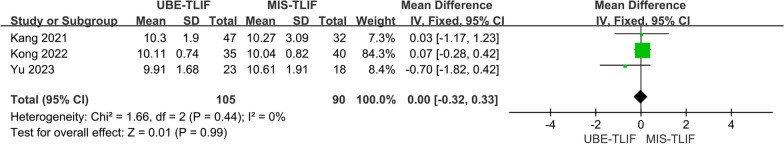
Forest plot for postoperative intervertebral disk height

A total of 3 studies both reported the intervertebral disk height (IDH) [16, 20, 25]. A total of 195 patients were included, including 105 patients in the UBE-TLIF group and 90 patients in the MIS-TLIF group. Heterogeneity between the two groups was low (*P* = 0.44, *I*^2^ = 0%), and Meta-analysis was performed using a fixed-effect model. The results of the analysis showed no statistically significant difference in the height of intervertebral disk height (IDH) between the two groups. (MD = 0.00; 95% CI (− 0.32, 0.33); *P* = 0.99, Fig. 16).

### CRP level on postoperative day 5

**Fig. 17:**
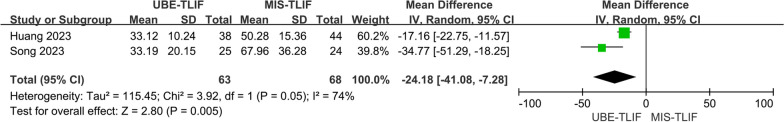
Forest plot for the CRP level on postoperative day 5

A total of 2 studies both reported the CRP level on postoperative day 5 [23, 24]. A total of 131 patients were included, including 63 patients in the UBE-TLIF group and 68 patients in the MIS-TLIF group. Heterogeneity between the two groups was low (*P* = 0.05, *I*^2^ = 74%), and meta-analysis was performed using a random-effect model. The results of the analysis showed no statistically significant difference in the CRP level on postoperative day 5 between the two groups. (MD = − 24.18; 95% CI (− 41.08, − 7.28); *P* = 0.005, Fig. 17).

### Heterogeneity analysis and publication bias

**Fig. 18:**
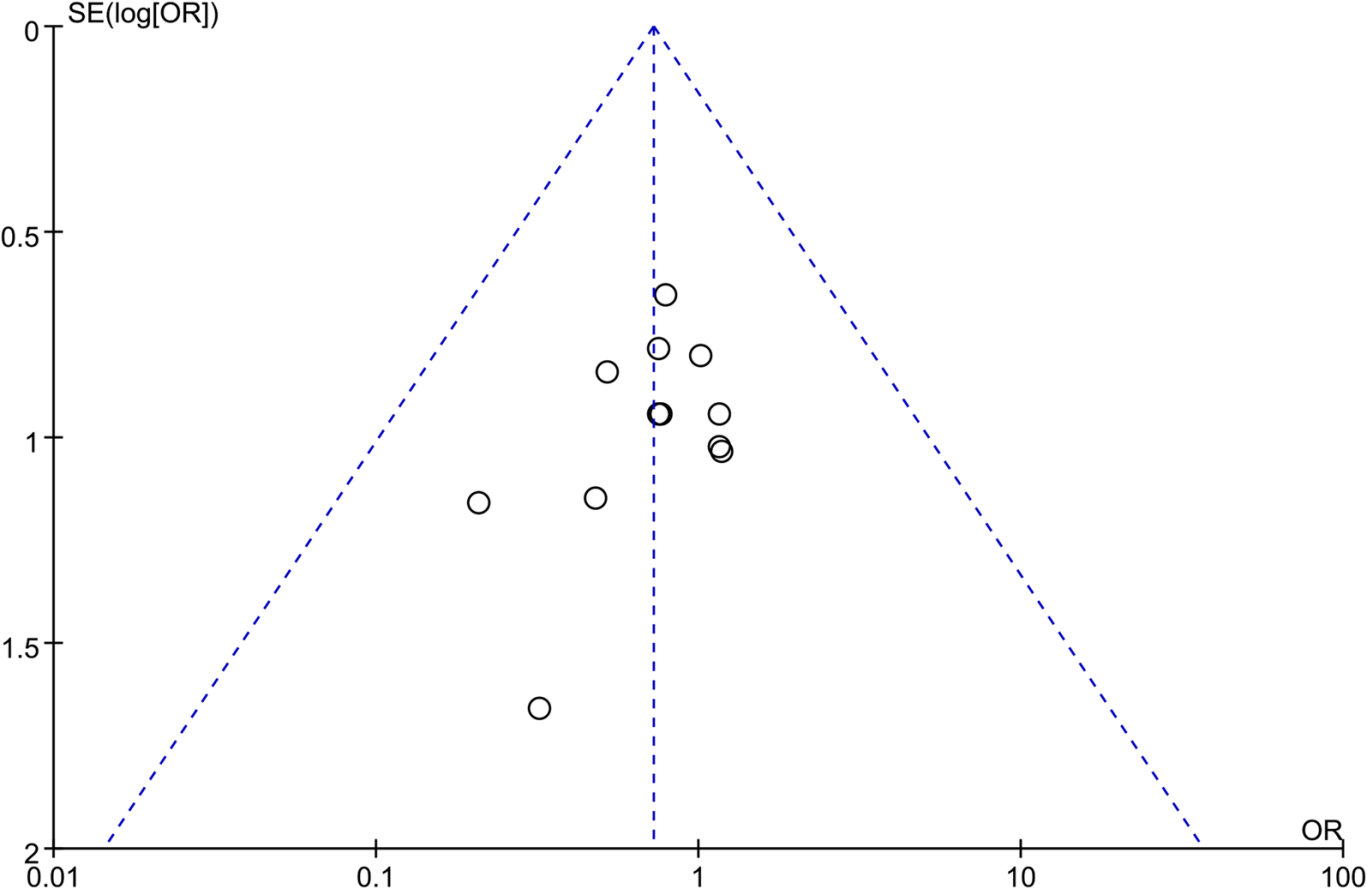
Funnel plot analysis for complication rate

Meta-analysis results of this study showed high heterogeneity in operative time, intraoperative blood loss, postoperative drainage, duration of hospital stay, early postoperative low back pain VSA score and early ODI score. Sensitivity analysis was performed, and a random-effect model was used to eliminate the effect of partial heterogeneity at *I*^2^ > 50%. Itemized exclusion of included studies, the heterogeneity remained high and little changed, indicating that the Meta-analysis result were relatively reliable and heterogeneity had little impact on the results of this study. Funnel plots were constructed to assess publication bias, and most studies are located in the upper part of the funnel plot and are largely symmetrical, indicating acceptable publication bias in our analysis (Fig. 18).

## Discussion

In recent decades, both UBE-TLIF and MIS-TLIF have become the most widely used minimally invasive techniques in the treatment of lumbar degenerative diseases, but their relative efficacy and safety have yet to be determined. By systematically comparing their outcomes in lumbar interbody fusion, our analysis suggested that UBE-TLIF would be a viable alternative to MIS-TLIF with better clinical outcomes with regard to earlier recovery. In present study, we included 12 outcome indications: including operative time, intraoperative blood loss, postoperative drainage flow, duration of hospital stay, total complication rate, modified Macnab grading criteria, fusion rate, VAS score, ODI score, postoperative CRP level, lumbar lordosis, and intervertebral disk height to compare the clinical efficacy of UBE-TLIF and MIS-TLIF.

In terms of surgical outcomes, the present study showed that there was a significant difference in the operative time between the two groups, which appeared to be longer in the UBE-TLIF group, this improvement may attribute to the following reasons: First, extensive searching for surgical landmarks, adequate decompression and percutaneous pedicle screw fixation under the endoscope requires increased operative time, thus increasing surgical risk. Second the control of intraoperative blood loss and adjustment of the clarity of the surgical field were essential factors affecting the operative time [11, 22]. Finally, the UBE technique also requires single-handed instrument handling which causes delicate procedures to become more complex to execute and complications such as dural tear and nerve root injury could occur, especially for less-experienced surgeons [15]. Learning curve studies by Chen [27] and Kim [28] showed that surgeons with experience in open and endoscopic spine surgery, respectively, approximately 24 or 34 cases reached a stable surgical level and were proficient in UBE technique to shorten the operative time. High heterogeneity existed in operative time, which mag due to the technique level of different surgeons and the utilization of different instruments during surgery. Intraoperative blood loss and postoperative drainage flow were significantly reduced in the ULIF group. There may be the following reasons: First, Continuous saline irrigation keeps the operative field clear, allows for compression of small vessel to stop hemorrhage [10, 29]. Second, Unlike MI-TLIF, UBE-TLIF does not require placement of a tubular retractor between the paravertebral muscles, thus reducing direct ischemic injury to the muscles [10, 13, 30]. (3) Less blood loss due to intraoperative magnification of the surgical field by the imaging system and hemostasis of soft tissue using radiofrequency electrodes. The high degree of heterogeneity in intraoperative blood loss may be due to the operator's control of bleeding during the course surgical procedure and the different methods of calculating blood in different studies. For example, gaze blood volume was calculated by weighing the gaze before and after surgery and dividing it by the density of the blood to convert to volume in Huang’s research [23], which was not taken into account in the calculation of blood loss in other studies. A total of 4 studies have reported that intraoperative blood loss was determined using aspirator suction, irrigation, and lavage volumes. Preoperative and postoperative Hematocrit was measured in Jiang’s study [19], intraoperative blood loss was calculated as a product of preoperative blood volume and Percentage of HCT loss. In the absence of infection, the peak postoperative CRP value is assumed to reflect the extent of tissue damage [31, 32]. CRP levels tend to peak on POD three and decrease rapidly to baseline between PODs ten and 14 [33]. UBE surgery is performed under continuous fluid irrigation, we could use radiofrequency ablation electrodes rather than electrocautery. This is a potential advantage in reducing surgical site infections because it has no surgical smoke and reduces wound contamination. Additionally, UBE-TLIF is less thermal injury to the paravertebral musculature and systemic inflammatory response than using electrocautery in MIS-TLIF. Huang [23] and Song [24] reported lower CRP levels in the UBE-TLIF group which also demonstrated that BE-LIF surgery can reduce damage to soft tissues and decrease the inflammatory response.

In terms of postoperative recovery effects, the UBE technique can short duration of hospital stay, and increase patient satisfaction. UBE is less stripping of soft tissues, while continuous saline irrigation reduces the production of inflammatory factors, which will benefit the patient in getting out of bed as soon as possible after surgery and functional recovery exercise. There is a high degree of heterogeneity in the duration of hospitalization, probably due to differences in social health insurance systems, individual physical difference and the type and length of conservative treatment, such as oral medication, physical therapy, and selective nerve root block. According to the modified MacNab criteria the excellent and good rates were 89.5% in UBE-TLIF and 87.8% in MIS-TLIF at last follow-up, there was no significant difference between the two groups. This indicates that both techniques are safe and effective.

Another major concern regarding the lumbar interbody fusion procedure is related complication. Although no significant difference was found in the overall complication rate of both UBE-TLIF (6.42%) and MIS-TLIF (8.14%), it is worth mentioning that UBE-TLIF seems to have a higher risk of potential nerve and vascular damage (Table 3). Zhu et al.’s [34] report showed that nerve tissue injury is considered to be the most significant complication of the UBE technique, with dural tears and nerve root injuries being the most common. For surgeons who perform the UBE technique in the early stage, cases with unilateral symptoms and mild degeneration were selected. The UBE technique can reduce tissue damage but requires skillful application of UBE techniques to avoid the adverse effects of prolonged surgery. The UBE has a reportedly good decompression effect without affecting the surgical safety, which maybe an alternative to conventional microsurgical decompression [35].

Pain improvement in the back and leg after lumbar spine surgery is also a critical outcome for deciding the surgical technique. Intraoperative dissection and paraspinal muscle retraction cause atrophy and denervation of subsequent muscles, thus increasing the possibility of postoperative low back pain. Heemskerk et al. [36] have reported that paravertebral muscle damage during surgery is closely related to the pressure of muscle retraction and duration of the retractor. The MIS-TLIF technique was performed using a Quadrant retractor system, and the surgery was completed with direct vision assisted by a channel. To ensure the surgical field, overstretching of the paravertebral muscles is usually unavoidable and can easily lead to local muscle ischemia and postoperative low back pain. Unlike MIS-TLIF, UBE-TLIF does not require placement of a tubular retractor between the paraspinal muscle. UBE technique establishes the observation and operation channels on the trigone of the multifidus muscle, which completes decompression of the spinal canal and intervertebral fusion. These two independent channels do not interfere with each other, and offer high flexibility and a wide field of view, which can effectively decrease traction and injury of the paravertebral muscles. In this study, the UBE-TLIF group VAS scores for low back pain and ODI scores were considerably lower 1 month after surgery than those of the MIS-TLIF group, indicating that the UBE-TLIF technique can effectively reduce the damage to the paravertebral muscles and the destruction of bone tissue, relieve postoperative low back pain and accelerate postoperative recovery. The effect of decompression is associated with the degree of recovery from leg pain after spinal surgery. Our study shows no significance difference in the VAS scores for leg pain from 1 month and 1 year after surgery. The UBE technique involves the use of an endoscope, which can be advanced adjacent to the lesion tissue and even into the lateral recess or intervertebral foramen. A smaller distance from the lesion improves the safety of surgical decompression and reduces the degree of laminectomy and better preservation of the facet joints. VAS and ODI are subjective metrics, and heterogeneity stems mainly from individual differences, with patients having different pain tolerances.

Radiological outcomes are the primary outcome measures used when deciding the surgical techniques for lumbar fusion surgery, where several factors are considered, including restoration of disk height, achievement of lordosis angle, and fusion rate. There was no significant difference between the two groups in terms of LL, IDH and fusion rate. Fusion rate is one of the most critical outcomes in evaluating efficacy, most of the literature reports that anterolateral and flexion–extension plain radiography and computed tomography (CT) were performed, and fusion rates were assessed by radiologists according to the Bridwell grading system, with grades I and II defined as spinal fusion. Some studies have reported that continuous saline perfusion flushing of the fusion bed within the intervertebral space during BE-LIF surgery, which leads to decreased blood supply and osteogenic factors, may lead to decreased fusion rates [16, 37]. In present study, the fusion rate was 88.6%% in the UBE-TLIF group and 88.4% in the MIS-TLIF group, both groups achieved good fusion rates and did not differ. The fusion rate at 1 year after surgery confirmed the non-inferiority of BE-TLIF compared to MT-TLIF. Additionally, preparation of the endplate implant bed is important in intervertebral interbody fusion and endplate bleeding is a sign of adequate preparation. Conventional spinal fusion procedures such as MIS-TLIF, rely on manual handling of the endplates, which may result in insufficient handling of the cartilaginous endplates or damage to the bony endplates [17]. Endoscopic procedures allow the operator to directly visualize and manipulate the endplate, establishing a favorable environment for the next implant fusion procedure.

### Limitation

This study still has some limitations: (1) the number of studies included in this Meta-analysis is small and they are all retrospective studies with a relatively low level of evidence; (2) the follow-up time is about 1 year and there is a lack of long-term controlled studies of clinical efficacy; (3) almost all necessary outcomes were included and analyzed in this study, but some secondary outcomes could not be assessed in this study because of insufficient inclusion. Therefore, large samples and multicenter controlled clinical studies are needed, especially high-quality RCTs and long-term follow-up results in confirming the advantages of UBE-TLIF in spine surgery.

## Conclusion

Based on our analysis, we conclude that UBE-LIF has more advantages in in terms of reduced intraoperative tissue injury and rapid postoperative recovery. Meanwhile, UBE is a new technique that can achieve the similar clinical results and does not produce more serious complications as MIS-TLIF. Therefore, UBE-TLIF is more beneficial to patients and offers a new and viable option for spine surgeons to perform lumbar fusion surgery. Further high-quality randomized controlled studies are needed to further confirm these results.

### Supplementary Information

**Additional file 1:** PRISMA checklist of Systematic review and Meta-analysis.

## Data Availability

The present study is a review and analysis of previously published literature. All relevant data and materials are reported within this manuscript, additional information will be available upon request to the corresponding author.

## Abbreviations

UBE-TLIF: Unilateral biportal endoscopic transforaminal lumbar interbody fusion
MIS-TLIF: Minimally invasive transforaminal lumbar interbody fusion
LDD: Lumbar degenerative disease
VAS: Visual analog scale
ODI: Oswestry Disability Index
LL: Lumbar lordosis
IDH: Intervertebral disk height
DVT: Deep vein thrombosis
NOS: Newcastle–Ottawa Scale
MD: Mean difference
CI: Confidence interval
PLIF: Posterior lumbar interbody fusion
CRP: C-reactive protein
RCT: Randomized controlled trial
HCT: Hematocrit
